# Silver Nanoclusters Encapsulated into Metal–Organic Frameworks for Rapid Removal of Heavy Metal Ions from Water

**DOI:** 10.3390/molecules24132442

**Published:** 2019-07-03

**Authors:** Pengfei Zhuang, Peng Zhang, Kuo Li, Beena Kumari, Dan Li, Xifan Mei

**Affiliations:** 1 Department of Basic Science, Jinzhou Medical University, Jinzhou 121001, China; 2 Department of Chemistry, Indian Institute of Technology Gandhinagar, Ahmedabad 382355, India

**Keywords:** nanoclusters, metal-organic frameworks, heavy metal ions

## Abstract

Metal nanomaterials have been reported as effective absorbents for the removal of pollutants in the water system, but the release of ions from these nanomaterials brings another concern. Herein, silver nanoclusters (AgNCs) were encapsulated in porous metal-organic frameworks of ZIF-8 (MOF-AgNCs). Compared to AgNCs, the release of Ag^+^ significantly decreases from MOF-AgNCs, indicating that the product presents a lower threat to the environment. The MOF-AgNCs were employed for the rapid removal of heavy metals, such as Pb^2+^ and Mn^2+^, from water. The mechanism and removal efficiencies were investigated.

## 1. Introduction

Environmental pollution in the form of water contamination is one of the most dangerous threats to human health. For instance, if large amounts of heavy metal ions are present in drinking water, the accumulation will affect multiple functions of the body [1]. Thus, efforts have been made to find promising materials to remove heavy metal contaminants from water [2]. Among the available strategies, nanoparticles (NPs) as absorbents show high removal efficiency [3,4,5]. This is because the NPs not only show low toxicities, but the large surface area enables an efficient absorption capacity [6]. In recent years, metal nanoclusters (NCs) have emerged as promising materials for environmental applications [7]. NCs are composed of a small number of atoms with sizes (<2 nm) smaller than traditional NPs [8]. Thus, they have even larger surface areas than NPs. These materials are promising to be used for green applications [9]. In particular, silver NCs (AgNCs) are excellent as cost-effective absorbents. Nevertheless, the drawback of these AgNCs is their susceptibility to oxidization [10]. This will facilitate the release of Ag^+^ and may affect the environment.

**Scheme 1:**
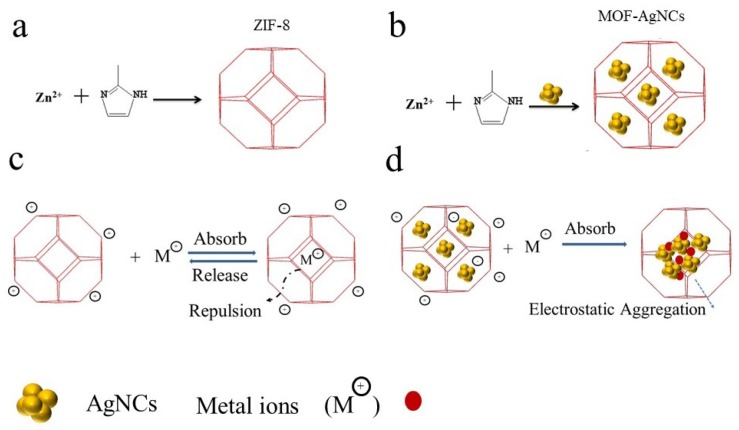
Schematic of the formation of ZIF-8 (**a**); encapsulation of AgNCs in metal-organic frameworks (**b**); the absorption of Mn^2+^ and Pb^2+^ by ZIF-8 (**c**) and MOF-AgNCs (**d**).

Metal-organic frameworks (MOFs) have attracted significant interest for the storage of different agents recently [11]. Zeolitic imidazolate frameworks (ZIFs), such as ZIF-8, are some of the most versatile MOFs with a large surface area and ultrahigh porosity [12,13,14]. MOFs have been employed as absorbents for the removal of heavy metal ions [15]. The high porosity of MOFs provides a path for the metal ions to penetrate and exit the pores. AgNCs have a tendency to co-precipitate with heavy metal ions. After encapsulating AgNCs in ZIF-8, the composite shows both the absorption and co-precipitation ability. For instance, when heavy metal ions are absorbed by MOF, the large size precipitates form due to the co-precipitation of heavy metal ions and AgNCs inside the ’cages’. Later, these large precipitates of heavy metal ion and Ag^+^ fails to penetrate out of the pores of ZIF-8. Thus, the synergistic effects of encapsulation of AgNCs by ZIF-8 as an absorbent will improve the removal efficiency of heavy metal ions. Herein, dihydrolipoic acid (DHLA) protected AgNCs were encapsulated in ZIF-8 to yield MOF-AgNCs nanocomposites (Scheme 1). In a MOF-AgNC composite, the stabilized AgNCs significantly suppress the release of Ag^+^. The prepared nanocomposites were investigated for the removal of typical heavy metal ions such as Mn^2+^ and Pb^2+^. Compared to ZIF-8, a higher removal efficiency of Pb^2+^ was obtained from MOF-AgNCs.

## 2. Results and Discussion

### 2.1. Characterization

**Figure 1:**
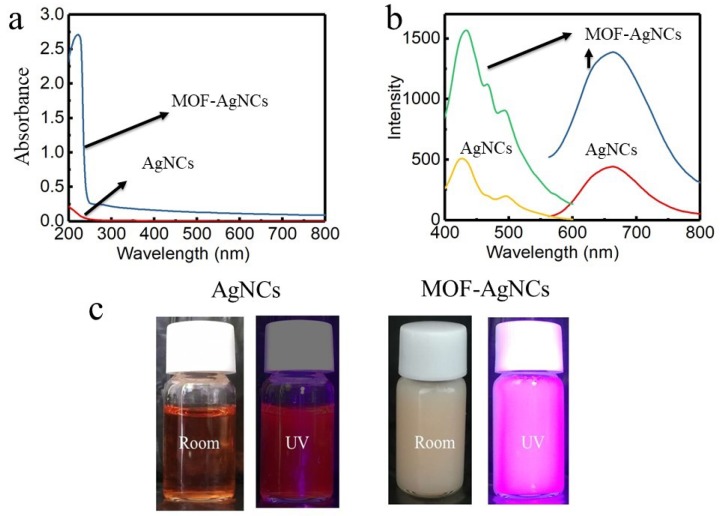
(**a**) UV-Vis, (**b**) fluorescence excitation (left), emission (right); (**c**) the corresponding photographs of AgNCs, and MOF-AgNCs at room and 365 nm UV light.

To test the successful encapsulation of AgNCs in ZIF-8, the optical properties of the AgNCs and MOF-AgNCs were investigated (see Figure 1). No obvious peak was observed in the absorption spectra of AgNCs with the current concentration (Figure 1a). On the other hand, the MOF-AgNCs showed the peak at ca. 220 nm in the UV–Vis spectrum. This peak is ascribed to the presence of ZIF-8. Moreover, for AgNCs an emission peak at ca. 660 nm was observed in the fluorescence emission spectrum (Figure 1b). The fluorescence intensity of MOF-AgNCs was much stronger than that for AgNCs. Along with this, it was observed that the MOF-AgNCs were brighter than AgNCs under the 365 nm UV light (Figure 1c). The phenomena are similar to the previously reported work for the encapsulation of NCs by MOFs [12]. The mechanism of fluorescence enhancement of MOF-AgNCs is described in Figure S1. During the excitation, the excited state rapidly relaxes to the first singlet excited state (S1) by the internal conversion process. The S1 state exhibits charge transfer behavior. At the same time, the intramolecular vibrations and rotations of the ligand (DHLA) in the shell of AgNCs causes nonradiative relaxation. These non-radiative emissions reduce the fluorescence intensity of independent AgNCs. The AgNCs are negatively charged because of DHLA. ZIF-8 are positively charged, when it combines with AgNCs the capping ligands (DHLA) were restricted to rotate due to the electrostatic interaction. This reduces the level of nonradiative relaxation of the excited states. As a result, the loss of energy is suppressed and the fluorescence is enhanced [12]. A similar fluorescence enhancement phenomenon is observed when the hydrogels, capsules, or nanosheets confine the ligand shell [16]. This strategy restricts the relaxation channels of the excited NCs and causes most of the electrons to return to the ground state through radiative decay rather than non-radiative decay.

**Figure 2:**
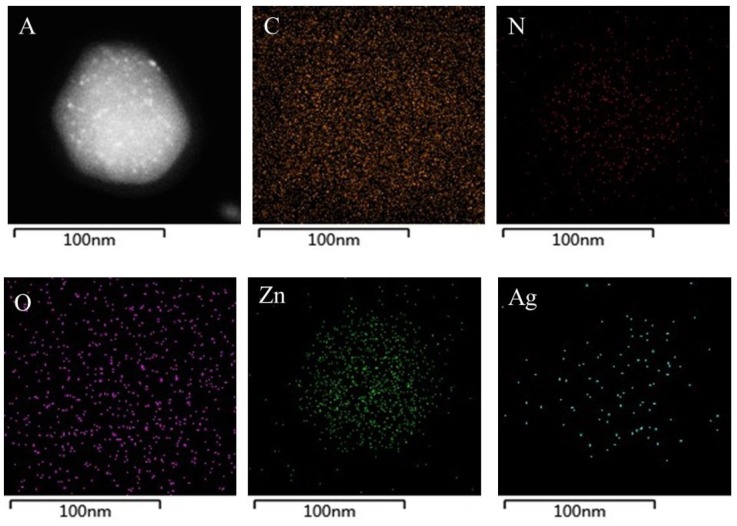
TEM images of MOF-AgNCs (**A**), and the corresponding elemental mappings for the nanocomposite (the element was marked on the images).

The successful fabrication of MOF-AgNCs was confirmed by TEM and Energy-dispersive X-ray spectroscopy mapping (EDS mapping) (see Figure 2). As indicated in Figure 2a, the small size NCs were observed. However, since ZIF-8 was relatively thick, the embedded NCs were not quite obvious. On the other hand, EDS-mapping clearly confirmed the presence of Ag and Zn elements. This indicates the coexistence of ZIF-8 and AgNCs.

### 2.2. Toxicity Investigation

**Figure 3:**
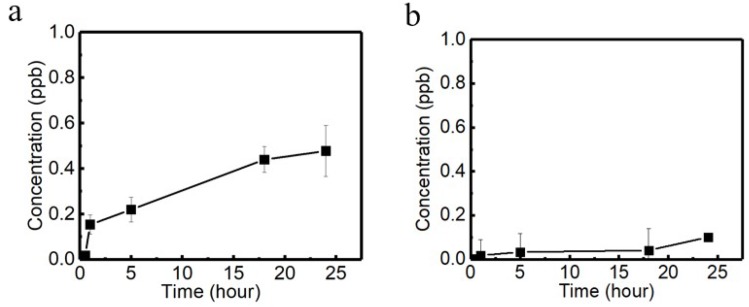
The concentration of Ag^+^ outside the dialysis tube as a function of time for AgNCs (**a**) and MOF-AgNCs (**b**) during the dialysis process.

The released Ag^+^ from AgNCs and MOF-AgNCs during the dialysis processes were studied as a function of time by using the same dosages (see Figure 3). For AgNCs, the released concentration for Ag^+^ kept increasing. After 24 h, the concentration of Ag^+^ was as high as ca. 0.5 ppb. On the other hand, the concentration of Ag^+^ was lower than 0.1 ppb in the presence of the same amounts of AgNCs. The release of Ag^+^ was dramatically decreased for the MOF-AgNCs. This indicates that the toxicity induced by Ag^+^ would be much lower by using MOF-AgNCs composite.

**Figure 4:**
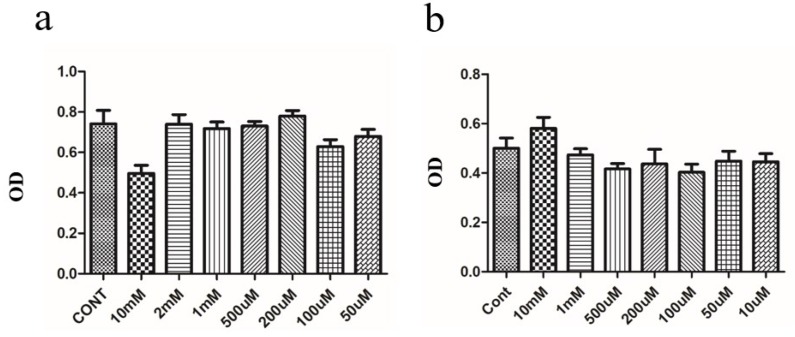
In vitro cell viability of HUVEC cells in the presence of different amounts of AgNCs (**a**) and MOF-AgNCs (**b**).

In order to compare the toxicity of the materials, the cell viabilities of human endothelial cells (HUVEC) cells were monitored (Figure 4). In contrast to AgNCs, MOF-AgNCs are less toxic at quite a higher concentration (10 mM) because of the reduced released Ag^+^. Another reason is the size of the MOF-AgNCs which is much larger than the AgNCs. The efficiency to penetrate the cell membrane is comparatively low. This indicates that MOF-AgNCs can be a promising green absorbent platform.

### 2.3. Removal of Heavy Metal Ions

#### 2.3.1. Mechanism Analysis

**Figure 5:**
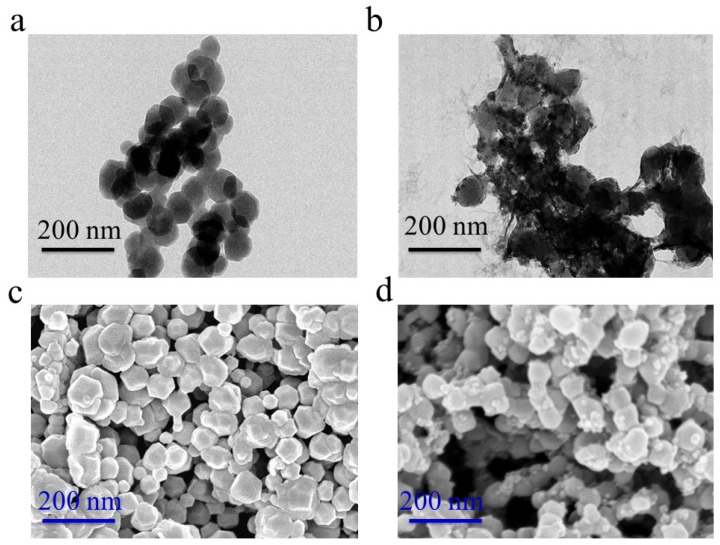
TEM (**a**,**b**), SEM characterizations (**c**,**d**) for MOF-AgNCs (**a**,**c**) and the products after the treatment of Mn^2+^ and Pb^2+^ (**b**,**d**).

To test whether MOF-AgNCs could be used to remove heavy metal ions, two typical ions including Mn^2+^ and Pb^2+^ were investigated. Since ZIF-8 are porous, the metal ions can easily penetrate inside the MOF. After that, the captured ions form large aggregates with AgNCs. Meanwhile, some metal ions were trapped on the surface of ZIF-8. The TEM (a, b), SEM (c,d) characterizations for the MOF-AgNCs before (a,c) and after the treatment of Mn^2+^ and Pb^2+^ (b,d) (Figure 5). It was observed that relatively larger size materials were present on the surface of ZIF-8 after the treatment (Figure 5b). The sizes were larger than AgNCs but smaller than ZIF-8. This was due to the agglomeration of the ultra-small size NCs with metal ions. Furthermore, the SEM characterization also indicates the presence of a new product (Figure 5d). Based on the investigation, it could be concluded that some NCs were leaking out and co-precipitated with the heavy metal ions, but they were still attached on the surface of the ZIF-8.

**Figure 6:**
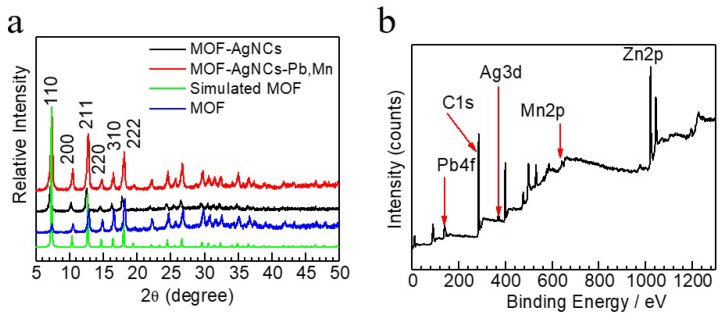
XRD patterns of ZIF-8, MOF-AgNCs and the product for the treatment of Mn^2+^ and Pb^2+^ (**a**); XPS survey of the product for removal of Mn^2+^ and Pb^2+^ (**b**).

In powder X-ray diffraction (XRD), the MOF-AgNCs before and after the treatment of Mn^2+^ and Pb^2+^ are displayed in Figure 6. The products exhibit almost identical XRD patterns to those of pure ZIF-8 (Figure 6a). This is in agreement with the previously reported work [17]. No diffraction patterns of Ag were observed for MOF-AgNCs. This is because of the poor crystallinity and low dosage of AgNCs in ZIF-8. The presence of Pb and Mn was hardly noticed by XRD characterization. Thus, the XPS characterization was performed (Figure 6b). The detailed spectrum of each element is demonstrated in Figure S2. The presence of Pb and Mn was clearly confirmed by an XPS survey. This indicates the successful absorption of Pb^2+^ and Mn^2+^ by MOF-AgNCs.

#### 2.3.2. Removal of Heavy Metal Ions

The fluorescence spectra for AgNCs and MOF-AgNCs were investigated in the absence and presence of the metal ions (Figure S3). It can be seen that Mn^2+^ quenches the fluorescence of both AgNCs and MOF-AgNCs. On the other hand, the fluorescence intensity of AgNCs was quenched, but the fluorescence of MOF-AgNCs was enhanced in the presence of Pb^2+^. AgNCs form conjugates or aggregates in the presence of Pb^2+^ and Mn^2+^ due to the formation of a chelation complex between functional groups −COO− and −OH on the surface of AgNCs [18]. The interactions between metal ions and AgNCs inside MOF-AgNCs are different. This is because both Mn^2+^ and Pb^2+^ have to penetrate inside the pore of ZIF-8 to interact with AgNCs. The diameter of Mn^2+^ is smaller than Pb^2+^, and it penetrates MOFs fast. Then, the larger size agglomerates of AgNCs form which quenches the fluorescence. On the other hand, Pb^2+^ slowly penetrates the pores of ZIF-8. As a result, soft aggregates form, which demonstrate the aggregation induced fluorescence enhancement effect. The fluorescence change indicates the interaction between AgNCs and the metal ions. This MOF-AgNCs show both absorption and precipitation ability. The interaction between MOF-AgNCs and the two metal ions were also investigated by high resolution TEM (Figure S4). It clearly illustrates that AgNCs significantly aggregate in the presence of Mn^2+^ (Figure S4a). However, it partly preserves their dispersed state by interacting with Pb^2+^. Thus, the fluorescence enhancement phenomenon is observed in the presence of Pb^2+^.

The removal ratio depends on the amounts of the absorbents. In order to compare their removal efficiency, much high concentration of both Mn^2+^ and Pb^2+^ are used for investigation, which is higher than the capacity of the absorbents. The results are shown in Figure S5. This confirms the encapsulation of AgNCs inside ZIF-8 improves the removal efficiency of Pb^2+^, but no significant improvement was observed for Mn^2+^.

**Figure 7:**
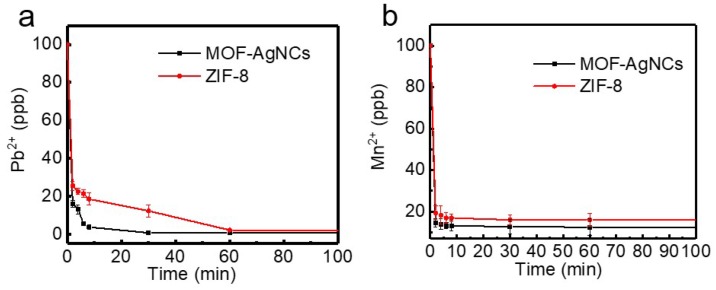
The removal of Pb^2+^ (**a**) and Mn^2+^ (**b**) in the presence of MOF-AgNCs and ZIF-8 as a function of time.

Normally, relatively low concentrations of Pb^2+^ and Mn^2+^ are present in natural water. The tap water was spiked with Pb^2+^ and Mn^2+^. After adding absorbents (MOF-AgNCs), the concentrations of the metal ions in the supernatants were measured (Figure 7). For Mn^2+^, the concentration was dropped from 100 ppb to lower than 20 ppb after half an hour. The MOF-AgNCs didn’t improve the removal efficiency significantly compared to ZIF-8. On the other hand, the concentration was reduced to less than 1 ppb quite fast for Pb^2+^ by MOF-AgNCs. Normally, it is difficult to reduce Pb^2+^ to trace level [19,20]. This indicates that the composite is quite promising to purify Pb^2+^ efficiently in aqueous system.

## 3. Materials and Methods

### 3.1. Materials

Zinc nitrate hexahydrate (ZnNO_3_. H_2_O), (±)-α-lipoic acid, 2-methyl imidazole, silver nitrate (AgNO_3_), and sodium borohydride (NaBH_4_) were purchased from Sigma-Aldrich (Shanghai, China). Other reagents and solvents were acquired from ALADDIN Reagent (Shanghai, China). Ultrapure water (18.2 MU) was used throughout the experiments.

### 3.2. Characterization

Fluorescence measurements were characterized using a F-97 fluorescence spectrophotometer (Shanghai Lengguang Technology, China). A JEM-2010 instrument (Jeol Ltd., Tokyo, Japan) equipped with an energy dispersive spectroscope (EDS) at 200 kV was used to investigate the transmission electron microscopy (TEM). Scanning electron microscopy (SEM) images were obtained using a Hitachi S-4800 FE-SEM instrument (Hitachi Chemical Company, Ltd., Tokyo, Japan). X-ray diffraction (XRD) measurements were investigated on a Bruker D8 FOCUS diffractometer using Cu Kα radiation (Billerica, MA, USA). UV-Vis spectroscopy was studied using a UV-1600 spectrometer (Shimadzu Scientific Instruments Inc., Columbia, MD, USA). Inductively coupled plasma mass spectrometry (ICP-MS) was studied by a Thermo Scientific instrument (Waltham, MA, USA).

### 3.3. Methods

#### 3.3.1. Synthesis of AgNCs

The AgNCs were synthesized through the reduction of AgNO_3_ in the presence of DHLA by NaBH_4_ with slight modification of a previous report. [21] Briefly, 60.8 mg of (±)-α-lipoic acid was dispersed in 15 mL of water. After that, 200 μL of 2 M NaOH was added to decompose (±)-α-lipoic acid to DHLA. The mixture was stirred until the colloid became transparent. Next, 200 μL of 100 mM AgNO_3_ was injected. Then, 1 mL of 1 M NaBH_4_ was added dropwise. The reaction mixture was stirred for 2–4 h until the fluorescence became stable.

#### 3.3.2. Synthesis of ZIF-8

ZIF-8 were synthesized according to a previous procedure. [22] 2.5 mmol ZnNO_3_ was dissolved in 10 mL of water. After that, 90 mL of 12.3 g of 2-methyl imidazole (0.15 mol) was combined. The samples were centrifugation under 10,000 rpm and washed with water three times.

#### 3.3.3. Synthesis of MOF-AgNCs

First, certain amounts (1 mL, 2 mL, 4 mL) of the as obtained AgNCs were mixed with 2 mL of 0.3 mol 2-methyl imidazole in water. Then, 2 mL (0.5 mmol) of Zn(NO_3_)_2_·6H_2_O was added. Then, the composite was washed three times with water. After that, the products were dried overnight in vacuum. Finally, the MOF-AgNCs with red-emitting fluorescence was obtained.

#### 3.3.4. Release of Ag^+^

2 mL of AgNCs and MOF-AgNCs with same weights were dispersed in 10 mL of water in a dialysis tube respectively. After that, the tubes were immersed in 100 mL of water with stirring for dialysis. Then, 1 mL water outside of dialysis tube was collected and diluted by 1% HNO_3_ at different interval of time. The concentration of Ag^+^ was determined by ICP-MS and calculated.

#### 3.3.5. Toxicity to animal cells

The in vitro study for the toxicity of the materials was evaluated by the viability of HUVECs by MTT assays. The cells were seeded at a density of 4 × 10^3^ per well in a 96-well plate and incubated overnight. Furthermore, the cells were exposed to the AgNCs and MOF-AgNCs with different concentrations (10 mM, 2 mM, 1 mM, 200 μM, 100 μM, 50 μM). The concentration is based on the silver element. The plates were incubated at 37 °C in 5% CO_2_ until a purple-colored formazan product was developed. After the media were carefully removed, 150 μL of dimethyl sulfoxide were used to dissolve the purple products. The absorbance at 490 nm was recorded by a microplate reader.

### 3.4. Removal of Mn^2+^ and Pb^2+^

In the mixture of 50 mL of 100 ppb of Mn^2+^ and Pb^2+^, 100 μg of MOF-AgNCs were dispersed. After a certain time, the precipitates were separated. The concentrations of the residual Mn^2+^ and Pb^2+^ in the supernatants were studied by ICP-MS.

## 4. Conclusions

The silver nanoclusters were successfully encapsulated by ZIF-8. The reduced release of Ag^+^ from MOF-AgNCs decreases the toxicity. The MOF-AgNCs show an excellent ability to remove Pb^2+^ in water. It is expected that the encapsulation of nanoclusters by MOF could be used to capture trace contaminants. This will open up a new horizon for wastewater treatment.

## Supplementary Materials

The following are available online at https://www.mdpi.com/1420-3049/24/13/2442/s1, Figure S1: Schematic illustration of the fluorescence enhancement of DHLA-AgNCs. S0, S1, S2 and IC represent the ground state, the first singlet excited state, the second singlet excited state, and the internal conversion process, respectively. Figure S2: XPS characterization of the product for removal of Mn^2+^ and Pb^2+^. Figure S3: Fluorescence emission spectra of AgNCs (**a**, **b**) and MOF-AgNCs (**c**, **d**) in the presence of 100 ppm of Mn^2+^ (**a**, **c**) and Pb^2+^ (**b**, **d**). Figure S4: HR-TEM for MOF-AgNCs in the presence of Mn^2+^ (**a**) and Pb^2+^ (**b**). Figure S5: Removal of Mn^2+^ (**a**) and Pb^2+^ (**b**) with different concentrations by same amounts of ZIF-8 and the encapsulation with relatively lower (MOF-AgNCs-1) and higher amounts of AgNCs (MOF-AgNCs-2).
