# Supplementary material for: Silver Nanoclusters Encapsulated into Metal–Organic Frameworks for Rapid Removal of Heavy Metal Ions from Water
Source: Molecules. 2019 Jul 3;24(13):2442. doi: 10.3390/molecules24132442 (PMC6651488; doi:10.3390/molecules24132442)
Supplement: Supplementary file 1 [file molecules-24-02442-s001.pdf]

Silver Nanoclusters Encapsulated into Metal–Organic Frameworks for Rapid Removal of Heavy Metal Ions from Water

Pengfei Zhuang [1], Peng Zhang [1], Kuo Li [1], Beena Kumari [2], Dan Li [1,*] and Xifan Mei [1,*]

[1] Department of Basic Science, Jinzhou Medical University, 121001, China
[2] Department of Chemistry, Indian Institute of Technology Gandhinagar, 382355, India.
* Correspondence: danli@jzmu.edu.cn (D.L.); meixifan1971@163.com (X.M.)

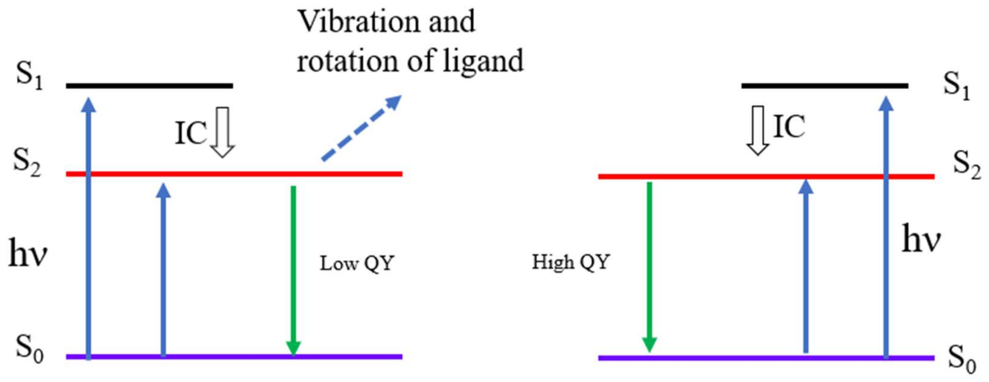

Figure S1. Schematic illustration of the fluorescence enhancement of DHLA-AgNCs. S_0, S_1, S_2 and IC represent the ground state, the first singlet excited state, the second singlet excited state, and the internal conversion process, respectively.

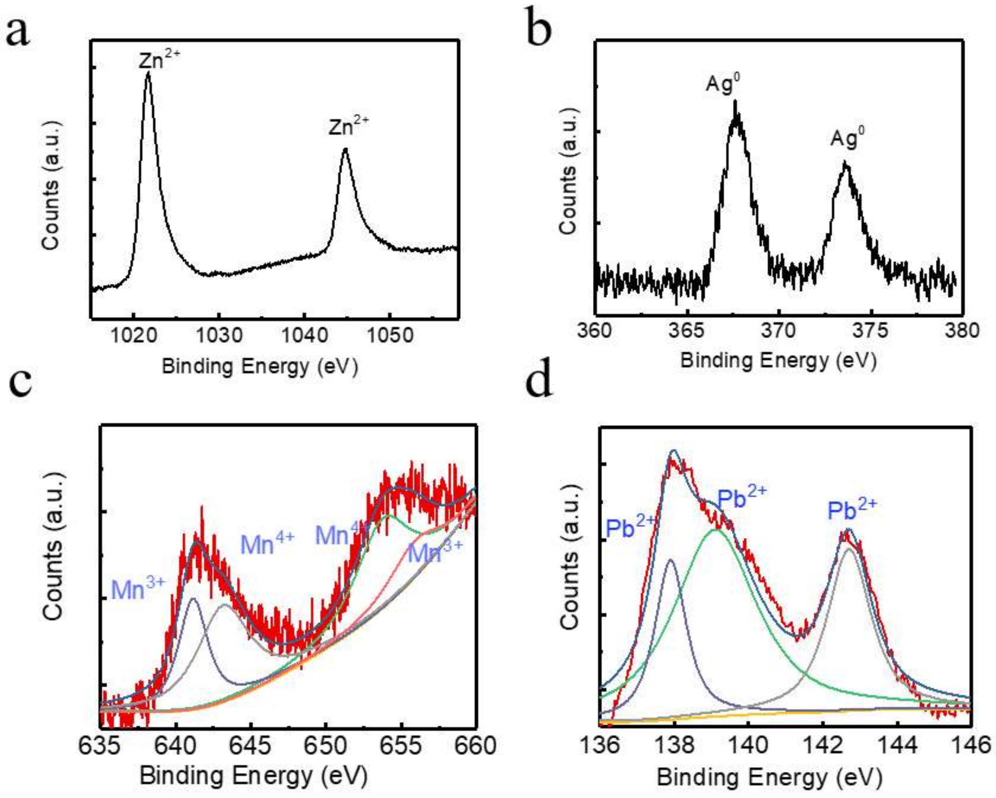

Figure S2. XPS characterization of the product for removal of Mn^{2+} and Pb^{2+}.

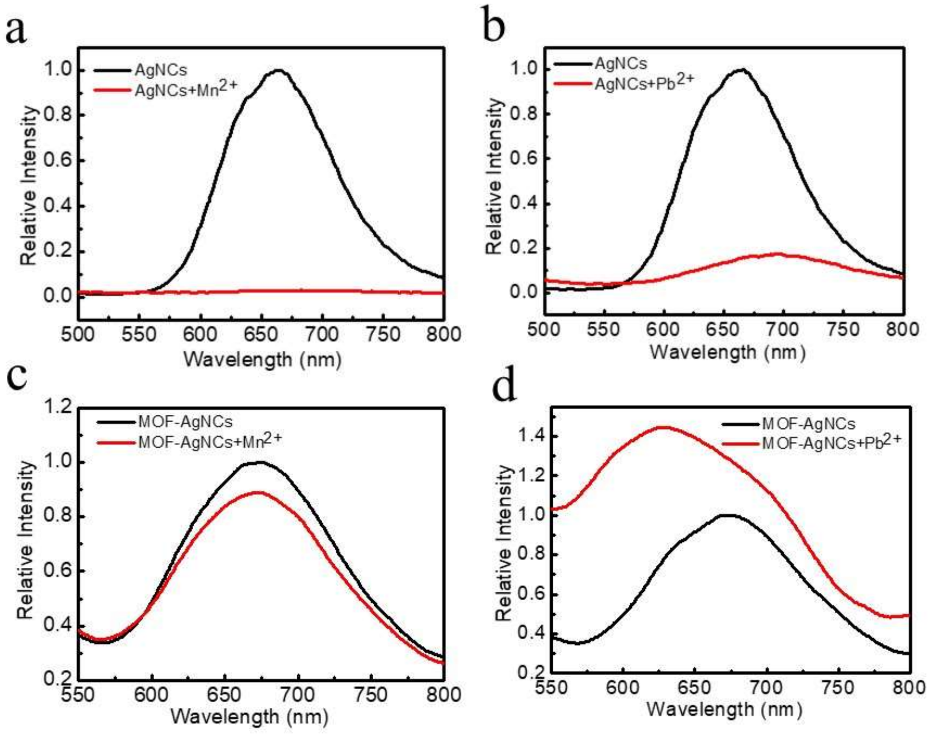

Figure S3. Fluorescence emission spectra of AgNCs (**a**, **b**) and MOF-AgNCs (**c**, **d**) in the presence of 100 ppm of Mn^{2+} (**a**, **c**) and Pb^{2+} (**b**, **d**).

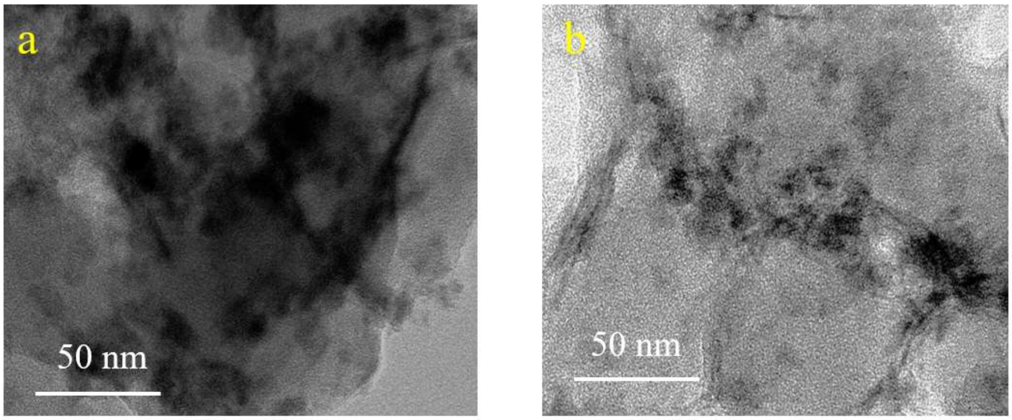

Figure S4. HR-TEM for MOF-AgNCs in the presence of Mn^{2+} (**a**) and Pb^{2+} (**b**).

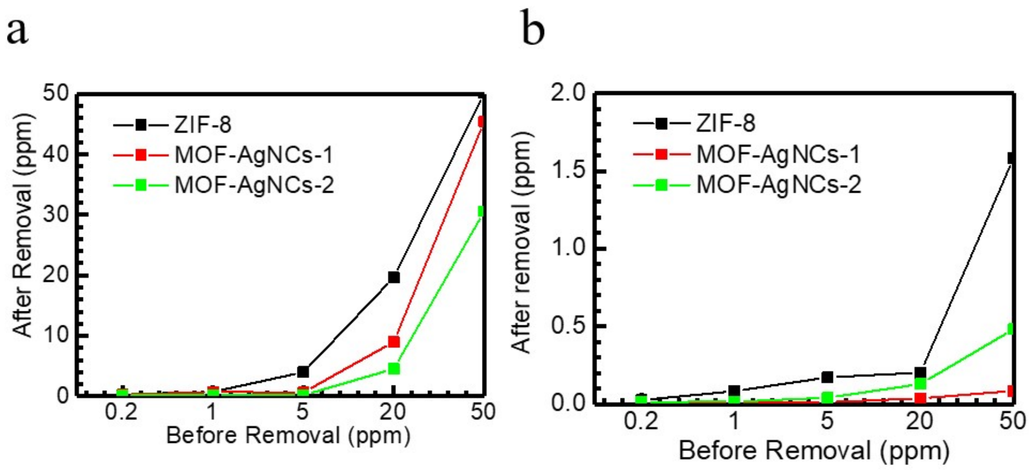

Figure S5. Removal of Mn^{2+} (**a**) and Pb^{2+} (**b**) with different concentrations by same amounts of ZIF-8 and the encapsulation with relatively lower (MOF-AgNCs-1) and higher amounts of AgNCs (MOF-AgNCs-2).
